# Development and Physicochemical Characterization of *Eugenia brejoensis* Essential Oil-Doped Dental Adhesives with Antimicrobial Action towards *Streptococcus mutans*

**DOI:** 10.3390/jfb13030149

**Published:** 2022-09-13

**Authors:** Maury Luz Pereira, Danyelle Cristina Pereira Santos, Carlos Alberto Mendes Soares Júnior, Tamyris Alicely Xavier Nogueira Bazan, Clovis Macêdo Bezerra Filho, Márcia Vanusa da Silva, Maria Tereza dos Santos Correia, Andres Felipe Millan Cardenas, Fabiana Suelen Figuerêdo de Siqueira, Edilausson Moreno Carvalho, Bruna Marin Fronza, Carolina Bosso André, Luis Claudio Nascimento da Silva, Lívia Câmara de Carvalho Galvão

**Affiliations:** 1 Postgraduate Program in Microbial Biology, CEUMA University, São Luís 65075-120, MA, Brazil; 2 Postgraduate Program in Dentistry, CEUMA University, São Luís 65075-120, MA, Brazil; 3 Department of Biochemistry, Federal University of Pernambuco, Recife 50751-310, PE, Brazil; 4 Department of Biomaterials and Oral Biology, School of Dentistry, University of São Paulo, São Paulo 05508-270, SP, Brazil; 5 Department of Restorative Dentistry, Operative Dentistry Division, Federal University of Minas Gerais, Belo Horizonte 30130-100, MG, Brazil

**Keywords:** anti-bacterial agents, biofilms, dental caries, *Eugenia*, polymers, *Streptococcus mutans*, volatile oils

## Abstract

Dental caries is a multifactorial, biofilm-dependent infectious disease that develops when detrimental changes occur in the oral cavity microenvironment. The antimicrobial and antivirulence properties of the essential oil obtained from the leaves of *Eugenia brejoensis* Mazine (EBEO) have been reported against Gram-positive and Gram-negative bacteria. Herein, the antimicrobial action of EBEO towards *Streptococcus mutans* is reported, along with the development and characterization of dental adhesives doped with. The minimum inhibitory concentration (MIC) and minimum bactericidal concentration (MBC) of EBEO were determined against *S. mutans*, while its toxicity was analyze using *Tenebrio molitor* larvae. EBEO (MIC and 10×MIC) was incorporated into the Ambar Advanced Polymerization System^®^ (Ambar APS), a two-step total-etch adhesive system (FGM Dental Group), and the antibiofilm action was evaluated. The reflective strength, modulus of elasticity, degree of conversion, and maximum rate of polymerization of each adhesive were also determined. The MIC and MBC values of EBEO against *S. mutans* were 62.5 µg/mL. The tested concentrations of EBEO were non-toxic to *T. molitor* larvae. The formation of *S. mutans* biofilms was significantly inhibited by EBEO and EBEO-coated resin discs (*p* < 0.05). Importantly, EBEO incorporation did not affect the mechanical and physicochemical properties in relation to oil-free adhesive version. EBEO showed strong antibacterial and antibiofilm activity against *S. mutans*, no toxicity effect against *T. molitor* larvae, and did not jeopardize the physical-chemical properties tested.

## 1. Introduction

Dental caries is a multifactorial, biofilm-dependent infectious disease that develops when detrimental changes occur in the oral cavity microenvironment [1,2]. It is characterized by an increase in the growth of cariogenic microorganisms (mainly, the genera *Streptococci*, *Lactobacillus*, *Actinomyces*, *Bifidobacteria*, *Candida*, *Veillonella*, and *Prevotella*) [3,4,5]. These pathogens are highly efficient at converting carbohydrates into organic acids, causing the demineralization of dental tissues [6]. The risk factors for dental caries involve poor oral hygiene, xerostomia, bad eating habits, food disorders, a lack of preventive dental care, and the alteration of the oral microbiota [7].

Among the microorganisms present in the oral cavity, *Streptococcus mutans* is the most related to the appearance of primary and secondary carious lesions and is therefore considered the main etiological agent of dental caries [8,9]. *S. mutans* can colonize tooth surfaces and form biofilms without the presence of glucose. In fact, *S. mutans* is the main producer of extracellular matrix (EM) in dental biofilm due its encoded glucosyltransferases, which enable the production of extracellular polysaccharides, promoting the adhesion of other microorganisms [5,8,10]. *S. mutans* can also metabolize a wide variety of carbohydrates to produce acids. Moreover, *S. mutans* can survive in low pH environments. Together, these characteristics are key virulence factors for *S. mutans* in dental caries [9,11,12]. There are also several virulence mechanisms that facilitate the survival and selection of *S. mutans* as a dominant strain under stressful conditions, such as dramatic pH changes, osmolarity differences, poor nutrient availability, and the presence of reactive oxygen species [9].

Recurrent (or secondary) caries, characterized by the recurrence of infection in restored teeth, are among the main causes of restoration replacement [13]. Thus, the association of antimicrobial agents with adhesive systems or composite resins is useful for preventing secondary caries [14,15]. The main advantage of incorporating an antimicrobial agent into the materials is the decrease in bacterial colonization, reducing the acid production, which causes dental demineralization, thus reducing the likelihood of restoration replacement [16,17]. Moreover, some studies have reported that the incorporation of antimicrobial agents, such as chlorhexidine, doxycycline, silver nanoparticles, monomers based on quaternary ammonium, or even isolated from natural products as apigenin and *tt*-farnesol, may benefit restorative procedures [17,18,19,20,21,22].

The essential oils (EO) are examples of antimicrobial agents incorporated in adhesive systems [23,24,25]. They are defined as complex mixtures of different chemical compounds (20–80 different molecules) that have attracted attention due their unique functional properties, for instance, they that can interact with various bacterial targets, such as membranes, protein synthesis, and efflux pumps, as well as virulence-related targets, such as biofilm formation [26,27,28].

The plants from the *Eugenia* genus are known to bear an essential oil, with several pharmacological potentials such as antioxidant, antitumoral, and antimicrobial activities [29]. In 2008, a novel species of *Eugenia* was discovered in the wetlands of the Pernambuco (Brazil), a place popularly known as “brejos”. Based on the location of its first description, this plant was named *Eugenia brejoensis* Mazine [30]. The essential oil purified from this plant (EBEO) has been found to exhibit potential larvicidal and antimicrobial effects against gram-positive bacteria [31,32,33]. EBEO is mainly composed of sesquiterpenes, such as δ-cadinene, β-caryophyllene, α-muurolol, α-cadinol, and bicyclogermacrene [33].

To develop new dental materials with better physical, chemical, and biological properties, with the aim of increasing the longevity of restorations and avoiding secondary caries, the present study evaluated the antibacterial and antibiofilm effects and toxicity of the essential oil derived from *E. brejoensis* Mazine (EBEO), along with the antibacterial and physicochemical properties of a commercial dental adhesive doped with EBEO at different concentrations. The null hypotheses were that EBEO (1) would not present a bactericidal and antibiofilm effect; (2) would not present toxicity effect; (3) when added to a dental adhesive would not present antibiofilm effects; and (4) when added to a dental adhesive would not affect it physical-chemical properties.

## 2. Results

### 2.1. EBEO Has Antibacterial Activity against Streptococcus mutans

Microdilution assays were performed to the minimum inhibitory concentration (MIC) and minimum bactericidal concentration (MBC) of EBEO against *S. mutans*. The EBEO had a MIC of 62.5 µg/mL. The MBC of EBEO coincided with the MIC, resulting in a MBC:MIC ratio of 1, which indicated a bactericidal effect.

### 2.2. EBEO Inhibits the Formation of Streptococcus mutans Biofilm

**Figure 1:**
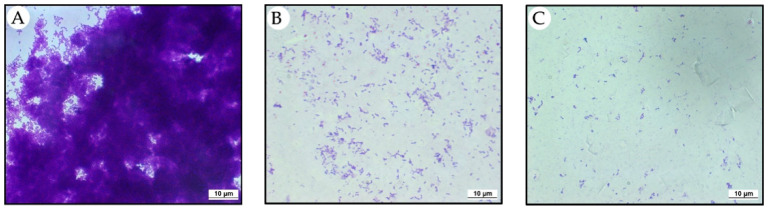
Biofilm of *Streptococcus mutans* on circular coverslip. (**A**) CON. (**B**) EBEO at 62.5 µg/mL (MIC). (**C**) EOEB at 625 µg/mL (10 × MIC). Biofilm marked with crystal violet appear in purple.

The anti-biofilm action of EBEO was evaluated in an assay using circular glass slides. In the control group, in which the biofilm was formed without of EBEO interference, biofilm production by *S. mutans* was abundant (Figure 1A). In groups where EBEO was used at MIC (62.5 µg/mL) and 10 × MIC (625 µg/mL), biofilm inhibitions were observed. The results were proportionally greater at the highest used concentration of EBEO (Figure 1B,C).

### 2.3. EBEO Is Non-Toxic towards Tenebrio molitor Larvae

**Figure 2:**
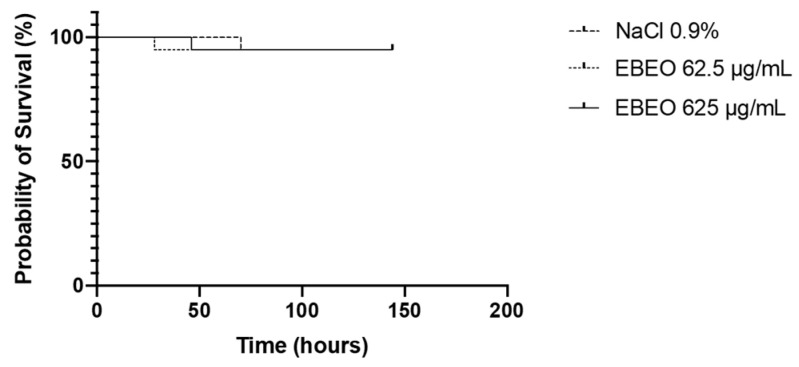
Percent survival of *Tenebrio molitor* larvae exposed to EBEO at MIC and 10×MIC concentrations. Kaplan–Meier death curves were plotted and estimates of differences in survival were compared using a log-rank test. Curves with the same pattern indicate no significant difference.

The toxicity of EBEO at MIC and 10 × MIC was analyzed in *T. molitor* larvae. Treatment with both EBEO concentrations did not show any toxicity to these larvae, resulting in a survival curve similar to that of the larvae inoculated with 0.9% NaCl (control group) (Figure 2).

### 2.4. Inhibition of Streptococcus mutans Biofilm Formation on EBEO-Coated Resin Discs

**Figure 3:**
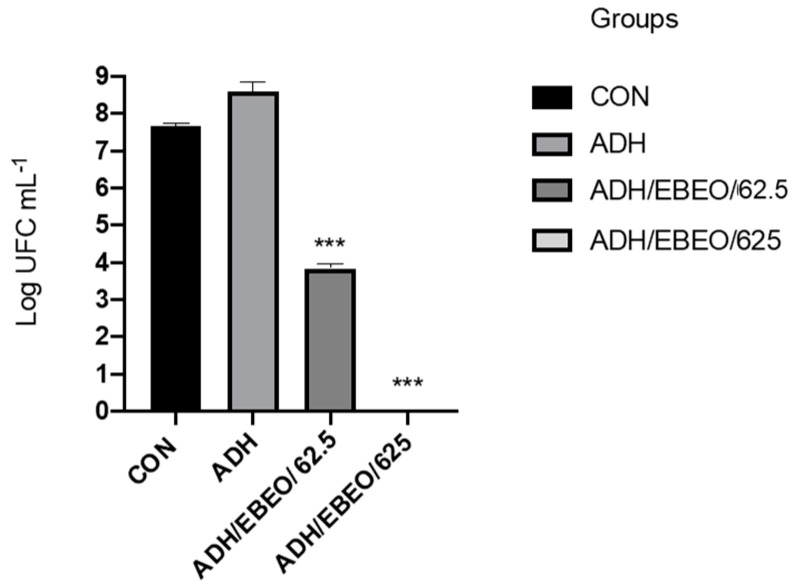
Viability of *S. mutans* in wells containing composite resin discs with and without dental adhesives tested. (***) Indicate significant difference from CON group.

Subsequently, the EBEO at MIC and 10 × MIC was used to modify the dental adhesives (Ambar APS). These adhesives were used to coat Resin composite discs (RCDs) with the following dimensions: 6 mm diameter and 1 mm thickness. The inhibition *S. mutans* biofilm formation was evaluated using composite resin discs either coated or not coated with adhesives containing EBEO. The results showed that the CFU from wells containing RCD without adhesive (CON) and RCD + adhesive groups (ADH) were in the order of 10^8^ CFU/mL. On the other hand, the CFU from RCD + adhesive with EBEO at MIC (62.5 µg/mL; ADH/EBEO/62.5) was reduced by more than half compared to the control. Finally, no bacterial growth was observed in wells that contained RCD + adhesive with EBEO 10×MIC (625 µg/mL; ADH/EBEO/625) (Figure 3).

The antibacterial tests performed indicate a bactericidal effect of EBEO and *S. mutans* antibiofilm activity when biofilm was treated with EBEO or grown on top of adhesive-doped EBEO.

### 2.5. Flexural Strength (FS) and Elastic Modulus (E)

The incorporation of EBEO at different concentrations (MIC and 10×MIC), into the adhesive did not reduce the FS and E. However, increases in the values of FS and E were observed when EBEO was incorporated at the MIC (Table 1).

### 2.6. Degree of Conversion and Maximum Rate of Polymerization

The incorporation of EBEO into the dental adhesive at the two tested concentrations (MIC and 10×MIC) did not jeopardize the degree of conversion or maximum rate of polymerization (Table 2).

The physical-chemical properties tested indicate that the addition of EBEO in concentrations of 62.5 µg/mL and 625 µg/mL did not negatively affect the dental adhesive behavior. 

## 3. Discussion

In this study, the action of EBEO against *S. mutans* was evaluated and, based on the antimicrobial effects observed, dental adhesives were developed and characterized. Natural products have been classified according to MIC value as follows: products with MIC lower than 500 μg/mL are strong inhibitors, those with MIC between 600 and 1500 μg/mL are moderate inhibitors, and those with MIC above 1600 μg/mL are weak inhibitors [28,34,35]. Data from this study indicate that EBEO is a strong inhibitor of *S. mutans*, with observed MIC and MBC values of 62.5 µg/mL (<500 µg/mL). These data are in accordance with the strong inhibitory effect that was also observed when EBEO was tested against *Staphylococcus aureus*, with MIC values ranging from 8 to 516 µg/mL [32]. 

Because MIC and MBC values were first evaluated with *S. mutans* in its planktonic form, EBEO was subjected to further testing in a more complex environment. Cells organized in biofilms present greater structural complexity and are more resistant relative to the action of antimicrobial agents [3,14]. Therefore, in this study, the antibacterial effect of EBEO was evaluated in *S. mutans* biofilm when diluted in culture media at MIC and 10×MIC. The EBEO at MIC (62.5 µg/mL) and 10× MIC (625 µg/mL) presented a strong reduction in *S. mutans* biofilm over circular coverslips, characterizing the potential to inhibit *S. mutans* biofilm formation, rejecting the first null hypothesis. 

Invertebrate models, such as insect larvae, have been widely used in the initial prospection of antimicrobial products. Insects can be bred quickly at low cost without the need for specialized equipment, and many larvae have immune systems similar to those of mammals [36,37]. In this study, the EBEO antimicrobial activity against *S. mutans* in planktonic and biofilm forms, EBEO, was non-toxic in a toxicity model using *T. molitor* larvae, accepting the second null hypothesis. The survival of larvae inoculated with EBEO at MIC was 95%, with similar results when compared to the control group, suggesting little or no toxic effect of the components present in EBEO at MIC and 10×MIC in a larval model. Another study evaluated the toxicity of EBEO in larvae of *Galleria mellonella*, and the results corroborate the present study, in which no toxicity was found compared to a control group. In addition, protection was observed in larvae infected with *S. aureus* by EBEO [32]. These data confirm the potential therapeutic applicability of EBEO.

Following these positive results, both concentrations were incorporated into the dental adhesives. A statistically significant difference was also observed when EBEO was added to the dental adhesive for biofilm formation; the EBEO at MIC reduced the biofilm formation by 50%, whereas no bacterial growth was observed at 10×MIC, rejecting the third null hypothesis. This result suggests that even when incorporated into dental adhesives and light-cured, EBEO has antibacterial and antibiofilm properties, without its activity being mitigated by the polymer matrix.

The antibacterial and antibiofilm activities of EBEO against *S. mutans* can be explained by its chemical composition as EBEO is mainly composed of sesquiterpenes, such as, β-caryophyllene, α-muurolol, α-cadinol, and bicyclogermacrene [33]. In particular, the inhibitory actions of β-caryophyllene, δ-cadinene, and α-cadinol against planktonic and biofilm forms of *S. mutans* have been previously reported [38,39]. Although positive results were found regarding antibacterial and antibiofilm effects, restorative materials containing antimicrobial compounds should be indicated for those patients with high-caries risk. This recommendation relies on the long-term effects of these antibacterial agents on resident oral bacterial and on the clinical longevity of composite restoration due to antibacterial leaching or physical-chemical properties lowering over time [21].

In dentistry, essential oils and derivatives have been added to mouthwashes [40] and toothpaste [41] and have been used to modify resin composite [42] and dental adhesive systems [23,24,25,43]. The examples of essential oils used to modify dental adhesive include essential oil of tea tree (Terpinen-4-ol) [24] and *Butia capitata* Oil [23,25]. The addition of EBEO to dental adhesives did not affect the physicochemical properties of the modified adhesives, accepting the fourth null hypothesis. DG, Rp_max,_ FS, and E were evaluated, and the physicochemical properties tested remained stable under all concentrations. However, after the incorporation of EBEO at MIC into the dental adhesive, a small increase in its FS and E was observed, while the values for 10×MIC did not differ from those of the control group. The mechanical properties are more dependent on the crosslink density than on the degree of conversion itself [44]. Therefore, it is suggested that a lower concentration of EBEO favors the formation of these cross-links. However, this activity was not observed at a higher concentration, which was likely due to the complex composition of EBEO. Similarly, the self-etch *B. capitata* essential oil-doped experimental adhesives also presented antimicrobial effects in a microcosm biofilm without affecting the physicochemical properties tested excluding microtensile bond strength, which decreased after six months of storage [25].

For this reason, although promising results were obtained, future studies are still necessary to identify whether EBEO can affect the immediate and long-term dentin bond strength, the antibacterial and antibiofilm long-term effects, and the additional physicochemical properties of the dental adhesive. In addition, given the polymicrobial character of caries, another limitation of this study was the use of an in vitro bacterial mono-specie model. Thus, the excellent results obtained in vivo encourage studies with mammals in caries models with multispecies biofilm.

## 4. Materials and Methods

### 4.1. Essential Oil Obtention

EBEO was provided by the Laboratory of Natural Products of the Federal University of Pernambuco (Recife, Brazil). The procedures used for EBEO extraction and characterization are reported by Silva et al. (2015) [33].

### 4.2. Bacterial Suspension

*S. mutans* UA159 was maintained as a pure culture prior to use in the experiment. *S. mutans* was initially reactivated from its original culture; grown on brain heart infusion (BHI) agar plates; and incubated at 37 °C in a 10% CO_2_ atmosphere, for 18–24 h. Colonies were suspended in 5 mL of 0.89% NaCl solution until an absorbance of approximately 0.135 (λ = 660 nm) was reached, which is equivalent to a 1–2 × 10^8^ colony-forming unit (CFU)/mL [28].

### 4.3. Minimum Inhibitory Concentration (MIC) and Minimum Bactericidal Concentration (MBC)

EBEO was tested against *S. mutans* to determine its MIC and MBC. In this assay, the MIC of bacterial growth and the viability of *S. mutans* were determined using the microdilution method in sterile 96-well culture microplates [28]. 

EBEO was diluted with propylene glycol. The solution was serially diluted in BHI broth to obtain a final concentration in the range of 10.0 to 1000.0 µg/mL. Subsequently, the BHI broth was inoculated with a bacterial suspension to obtain a concentration of approximately 1–2 × 10^5^ CFU/mL. The plates were then read using a single-channel microplate reader (ASYA HITEH–UVM 340, Biochrom, Holliston, MA, USA) and incubated at 37 °C with 10% CO_2_ for 24 h. After incubation, absorbance at 625 nm was measured using a single-channel microplate reader. The MIC was considered the lowest concentration of EBEO at which there was no bacterial growth (absorbance reading lower than 0.05). Chlorhexidine digluconate (0.12%, final concentration) was used as the positive control, and the same vehicle and concentration were used in the preparation of the EO (25% propylene glycol) for the negative control group. The MBC was determined based on the results obtained in the MIC test, and the lowest concentration of EBEO at which no cell growth was observed on the inoculated agar surface was recorded for MBC (99.9% bacterial death). To determine the nature of the antibacterial effect of the EBEO, the MBC:MIC ratio was determined by considering the following criteria: when the MBC:MIC ratio was 1:1 or 2:1, the product was considered bactericidal; and when the ratio was greater than 2:1, the product was considered bacteriostatic [28].

### 4.4. Anti-Biofilm Assay on Circular Coverslip

To evaluate the antibiofilm activity of EBEO against *S. mutans*, 12-well microplates containing glass coverslips were used. For this, 200 µL of BHI culture medium, the bacterial inoculum at a concentration of 1 × 10^6^ CFU/mL, glucose at a final concentration of 1%, and EBEO at the MIC and 10×MIC were added in triplicate to obtain three groups:

Control Group: *S. mutans* + BHI + 1% Glucose (CON);
Group 2: *S mutans* + BHI + 1% Glucose + EBEO in MIC (EBEO 62.5);
Group 3: *S. mutans* + BHI + 1% Glucose + EBEO 10×MIC (EBEO/625).

Subsequently, circular coverslips measuring 13 mm in diameter were inserted into the wells using sterile forceps. The plates were incubated in 10% CO_2_ at 37 °C for 24 h. After this period, the circular coverslips were carefully removed from the plates, washed twice with saline solution, and fixed with 200 µL of methanol for staining with 0.1% crystal violet. Following staining, the coverslips were evaluated Zeiss^®^ Axio Imager fluorescence microscope (Zeiss, São Paulo, Brazil).

### 4.5. Toxicity in Tenebrio molitor Larvae

Larval stage insects were used for the EBEO toxicity assays. Groups of 20 larvae (sample size calculation recommended at least seven larvae, considering power of 0.95 and α = 0.05) [45], weighing 70–120 mg and without signs of melanization, were randomly selected and used for subsequent inoculation. A 50 μL Hamilton^®^ syringe was used to inject 5 μL aliquots of EBEO at MIC and 10×MIC, as well as 5 μL of saline (negative control) into the hemocoel of larvae through the last left proleg. After injection, larvae were stored at room temperature, and the appearance of melanization signs and survival were recorded at selected intervals for 7 days. Larvae were classified as dead if they did not exhibit any movement in response to touch. Kaplan–Meier death curves were plotted and estimates of differences in survival were compared using a log-rank test. Statistical significance was set at *p* < 0.05. 

### 4.6. Adhesive Formulation

The dental adhesive used in this study was the Ambar Advanced Polymerization System^®^ (Ambar APS), a 5th generation (two-step total-etch) light-curing adhesive system (FGM dental group, Joinville, Brazil). The composition of this adhesive, according to the manufacturer information, is shown in Table 3. The adhesive Ambar APS was used as a vehicle to incorporate EBEO. For this, 375 µL or 37.5 µL of EBEO solution (10 mg/mL) was diluted in 6 mL of the vehicle to obtain the adhesives with EBEO at 62.5 µg/mL (MIC) or 625 µg/mL (10×MIC), respectively. The formulations were prepared using sterilized materials, in a yellow room to avoid the polymerization reaction by blue light and mixed with a mechanical agitator.

### 4.7. Inhibition of Streptococcus mutans Biofilm Formation on EBEO-coated Resin Discs

Resin composite discs (RCDs) were obtained (Llis, FGM, EA1), following the pattern of a mold matrix with the following dimensions: 6 mm diameter and 1 mm thickness. The composite discs were covered with a thin layer of Ambar APS adhesive with or without the EBEO, standardizing 7 µL of the adhesive on each side of the composite disc and divided into four groups (n = 4; following sample size calculation with power of 0.95 and α = 0.05) [46]:

Control Group: RCD without adhesive (CON);
Group 2: RCD + adhesive (ADH);
Group 3: RCD + adhesive with EBEO at MIC (ADH/EBEO/62.5);
Group 4: RCD + adhesive with EBEO at 10×MIC (ADH/EBEO/625).

Four RCDs were first light-cured, and 7 µL aliquots of the adhesives were light-cured under the RCDs using an LED light-curing unit (Valo, Ultradent, Indaiatuba SP, Brazil) for 20 s on each side (1200 mW/cm^2^). The samples were placed in 96-well microplates containing the BHI culture medium, the bacterial inoculum at a concentration of 1 × 10^6^ CFU/mL, and glucose at a final concentration of 1%. The plates were incubated in 10% CO_2_ at 37 °C for 24 h. Following this period, aliquots of the culture medium were removed, diluted, and plated to count CFU/mL.

### 4.8. Flexural Strength (FS) and Elastic Modulus (E)

The FS and E of the dental adhesives with and without EBEO were measured. Bar-shaped specimens were obtained (12 mm × 2 mm × 2 mm) for all groups (n = 10; sample size calculation recommended at least four specimens, considering power of 0.95 and α = 0.05) using a standard stainless-steel mold [35]. The solvent was evaporated using an oil-free air jet for 10 s. The bars were light-cured, and the light cure LED units were set at a power of 1.200 mW/cm^2^ (Valo, Ultradent, South Jordan, UT, USA) for two overlapping exposure periods of 20 s each (20 s on the right side, 20 s on the left side). The bottom was also light-cured for an additional 20 s, and the samples were stored for 24 h under light-free, room-temperature, and dry conditions. A single operator performed all procedures. 

After storage, the specimens were tested using the three-point bending method on a universal testing machine (Instron model 5844, Instron Corp., Canton, MA, USA) at a crosshead speed of 0.5 mm/min. The FS was calculated as *FS = 3Fl/2bh^2^*, where *F* is the load at fracture (N), *l* is the span length (10 mm), and *b* and *h* are the width and thickness of the specimens, respectively (mm), and expressed as MPa. The E was determined from the slope of the initial linear part of the stress-strain curve and was calculated as *E = FI^3^/4bh^3^d*, where *F* is the load at some point on the linear region of the stress-strain curve; *d* is the slack compensated deflection at load *F*; and *l*, *b*, and *h* are the same as defined above and expressed as GPa.

Data were subjected to the Shapiro–Wilk normality test and homoscedasticity and analyzed using one-way analysis of variance (ANOVA) followed by the Holm–Sidak post-hoc test (α = 0.05). 

### 4.9. Degree of Conversion and Maximum Rate of Polymerization

The polymerization kinetics of adhesives with and without the incorporation of EBEO at different concentrations was monitored using Fourier transform infrared spectroscopy (FT-NIR, Vertex 70, Bruker Optics, Billerica, MA, USA) in specimens (n = 3; following sample size calculation with power of 0.95 and α = 0.05) of 5 mm diameter and 1 mm thickness between two glass slices. The solvent was evaporated for 10 s. The area of the aliphatic peak, corresponding to the carbon double bonds of the methacrylate group, centered at 6165 cm^−1^, was used to monitor the polymerization reaction in real time continuously for 6 min. The samples were light-cured for 20 s at an incident irradiance of 1200 mW/cm^2^ (Valo, Ultradent, South Jordan, UT, USA). The degree of conversion (DC) was calculated according to the formula: DC = (1 − final peak area/initial peak area) × 100%. The maximum polymerization rate (Rp_max_) was calculated through the maximum point at the first derivative from the “conversion × time” curve. Data were analyzed using one-way ANOVA (α = 0.05).

## 5. Conclusions

Within the limitations of this in vitro study, it can be concluded that (1) EBEO presented MIC and MBC of 62.5 µg/mL; (2) EBEO showed strong antibacterial and antibiofilm activity against *S. mutans*; (3) EBEO showed no toxicity effect against *T. molitor* larvae; (4) when incorporated into a dental adhesive, EBEO held its antimicrobial activity and did not jeopardize the physical-chemical properties of the adhesive tested; and (5) the innovative EBEO-doped dental adhesives may have great promise in the prevention of recurrent caries for high-risk caries patients.

## Figures and Tables

**Table 1:** Flexural strength and elastic modulus of adhesives tested.

Groups	FS (MPa)	E (GPa)
**Adhesive (control group)**	40.9 (±5.3) ^a^	0.16 (±0.01) ^a^
**Adhesive + EBEO (62.5 µg/mL)**	51.8 (±6.6) ^b^	0.20 (±0.02) ^b^
**Adhesive + EBEO (625 µg/mL)**	42.3 (±3.4) ^a^	0.16 (±0.01) ^a^

In each column, different superscript letters (^a, b^) indicate significant difference (*p* < 0.05).

**Table 2:** Degree of conversion (DC) and maximum polymerization rate (Rp_max_) of tested adhesives.

Groups	DC (%)	Rp_max_ (%/s)
**Adhesive (control group)**	94.6 (±3.1) ^a^	16.4 (1.5) ^a^
**Adhesive + EBEO (62.5 µg/mL)**	94.5 (2.2) ^a^	18.6 (2.3) ^a^
**Adhesive + EBEO (625 µg/mL)**	94.8 (0.5) ^a^	18.9 (0.9) ^a^

In each column, the same superscript letter (^a^) indicates no significant difference (*p* > 0.05).

**Table 3:** Adhesive composition.

	Components
**Ambar APS**	Urethane dimethacrylate glycerol dimethacrylate.
	2-Hidroxyethylmethacrylate
	Ethanol
	10-Methacryloyoxydecyl dihydrogen phosphate
	Ethyl 4-(Dimethylamino) Benzoate
	Adhesive monomer (under patent protection)
	Antioxidant (under patent protection)
	Camphorquinone

## Data Availability

Data available upon request.

## Sample Availability

Sample available upon request.
